# Development of Various *Leishmania* (*Sauroleishmania*) *tarentolae* Strains in Three *Phlebotomus* Species

**DOI:** 10.3390/microorganisms9112256

**Published:** 2021-10-29

**Authors:** Lucie Ticha, Barbora Kykalova, Jovana Sadlova, Marina Gramiccia, Luigi Gradoni, Petr Volf

**Affiliations:** 1 Department of Parasitology, Faculty of Science, Charles University, Vinicna 7, 12800 Prague, Czech Republic; barbora.kykalova@natur.cuni.cz (B.K.); jovana.sadlova@natur.cuni.cz (J.S.); 2 Department of Infectious Diseases, Unit of Vector-Borne Diseases, Istituto Superiore di Sanità, Viale Regina Elena 299, 00161 Rome, Italy; marina.gramiccia@iss.it (M.G.); luigi.gradoni@iss.it (L.G.)

**Keywords:** *Sauroleishmania*, *Leishmania tarentolae*, sand flies, *Phlebotomus*, experimental infections

## Abstract

*Leishmania* (*Sauroleishmania*) *tarentolae* is transmitted by reptile-biting sand flies of the genus *Sergentomyia*, but the role of *Phlebotomus* sand flies in circulation of this parasite is unknown. Here, we compared the development of *L.* (*S.*) *tarentolae* strains in three *Phlebotomus* species: *P. papatasi*, *P.* *sergenti*, and *P. perniciosus*. Laboratory-bred sand flies were membrane-fed on blood with parasite suspension and dissected on days 1 and 7 post blood meal. Parasites were measured on Giemsa-stained gut smears and five morphological forms were distinguished. In all parasite-vector combinations, promastigotes were found in Malpighian tubules, often in high numbers, which suggests that this tissue is a typical location for *L.* (*S*.) *tarentolae* development in sand flies. All three studied strains colonized the hindgut, but also migrated anteriorly to both parts of the midgut and colonized the stomodeal valve. Significant differences were demonstrated between sand fly species: highest infection rates, high parasite loads, and the most frequent anterior migration with colonization of the stomodeal valve were found in *P. perniciosus*, while all these parameters were lowest in *P. sergenti*. In conclusion, the peripylarian type of development was demonstrated for three *L*. (*S*.) *tarentolae* strains in three *Phlebotomus* sand flies. We suggest paying more attention to *Phlebotomus* species, particularly *P. perniciosus* and *P. papatasi*, as potential secondary vectors of *Sauroleishmania*.

## 1. Introduction

Protozoans of the genus *Leishmania* (Kinetoplastida: Trypanosomatidae) are causative agents of leishmaniases, neglected tropical diseases that affect millions of people worldwide. *Leishmania* parasites have digenetic life cycle, circulating between a wide range of reservoir hosts and phlebotomine sand flies (Diptera: Psychodidae). Members of this genus were recently divided into four subgenera: *Leishmania*, *Viannia*, *Sauroleishmania*, and *Mundinia* [1]. While the subgenera of *Leishmania* and *Viannia* have been intensively studied, little is known about *Sauroleishmania* and their life cycle.

*Sauroleishmania* was first described as a separate genus in 1973 [2], later the same subgeneric name was used to separate all reptiles-infecting species from those that infect mammals [3]. Subsequently, other species were placed into this genus and its definition was better constituted [4]. The phylogenetic position of *Sauroleishmania* was unclear for a long time, based on recent molecular data, it is now generally accepted that *Sauroleishmania* form a monophyletic group that belongs within the genus *Leishmania* [5]. The subgenera *Leishmania* and *Sauroleishmania* are sister-groups that divided relatively lately, and some studies suggest the hypothesis of the origin of *Sauroleishmania* parasites due to the adaptation of mammalian species to the reptile hosts [5,6,7]. Currently, 21 species belonging to the subgenus *Sauroleishmania* are described, including two unnamed species [5].

*Sauroleishmania* has been a neglected group of parasites so far as it was considered as non-pathogenic for mammals. They are regarded as parasites of reptiles and they have been repeatedly isolated from many different reptile species, mainly lizards of the families Agamidae, Gekkonidae, Iguanidae, Lacertidae, and Scincidae [8,9]. However, some members of this subgenus were able to infect, at least transiently, mammals or mammalian cells [10,11,12,13]. Recently, DNA of these parasites was detected in asymptomatic rodent [14], canine blood [15], or human blood [16]. These findings are raising questions about the current biosafety level of these parasites [7].

The mechanism of *Sauroleishmania* transmission remains unclear; it is considered that reptiles are infected by sand fly bite and/or by its ingestion [4,17]. Proven natural vectors are reptile-biting sand flies of the genus *Sergentomyia* [4,18]. On the other hand, the role of *Phlebotomus* sand flies in *Sauroleishmania* circulation remains unclear. Some *Phlebotomus* species occasionally feed on reptiles [8,19,20] and old studies reported that some *Sauroleishmania* parasites can develop late-stage infections in *Phlebotomus* sand flies [8,17,21]. Nevertheless, further research in this area is needed. *Sauroleishmania* development in sand flies is localized in the hindgut and thus is described as hypopylarian [22]. However, there are some older records of the anterior migration of these parasites in the sand fly gut [23,24,25], which indicates that some *Sauroleishmania* parasites might be transmitted by bite via the mechanism known for mammalian species [26].

*Leishmania* (*S*.) *tarentolae* is the most studied *Sauroleishmania* species. It was first isolated from a common wall gecko, *Tarentola mauritanica* [27,28,29]. In these geckos, amastigotes were found inside the monocytes [30]. The infection of *L.* (*S*.) *tarentolae* was also discovered in some other gecko species, namely *Cyrtodactylus kotschyi* [31] and *Tarentola annularis*, in which amastigotes were observed inside leucocytes [32]. However, DNA of *L*. (*S*.) *tarentolae* was recently detected in lacertid lizards, *Podarcis siculus* [15], suggesting that geckos might not be exclusive hosts for this *Sauroleishmania* species. In Italy, promastigotes typed as *Leishmania* (*S*.) *tarentolae* were isolated from *Sergentomyia minuta* in Calabria [18] and Rome provinces [33]; in both localities, the infection rates of *Se. minuta* females were relatively high (7.1% and 2.3%, respectively). In addition to *Se. minuta*, two other sand fly species were reported as potential vectors of *L.* (*S*.) *tarentolae*: *Sergentomyia antennata* and *Phlebotomus papatasi* [17,21,23]. Nevertheless, a detailed description of the development in sand flies is absent.

In this study, we compared the development of three *L.* (*S*.) *tarentolae* strains of different origin in three sand fly species of the genus *Phlebotomus*, with the focus on the localization of parasites in the sand fly gut and description of various morphological stages. The potential role of these sand fly species in the transmission of *Sauroleishmania* is also discussed.

## 2. Materials and Methods

### 2.1. Parasites and Sand Flies

Three *Leishmania* (*S*.) *tarentolae* strains of different origin were used for the experiments. RTAR/IT/1981/ISS21-G6c isolated from *Tarentola mauritanica* in Apulia and typed by Multilocus Enzyme Electrophoresis (MLEE) [29], RCYR/IT/1981/ISS24-CK3 isolated from *Cyrtodactylus kotschyi* also in Apulia and typed by MLEE [31] and IMIN/IT/2017/ISS3200-RM-5 isolated from *Sergentomyia minuta* in Latium and typed by ribosomal ITS1-PCR RFLP [33,34]. Originally, all strains were isolated in Evans’ modified Tobie’s medium, quickly cryopreserved after a few subinoculations, and maintained thereafter at the *Leishmania* cryobank of the Istituto Superiore di Sanità, Rome. For this study, recently thawed parasites were cultivated at 23 °C in SNB-9 blood agar [35] with Medium 199 (Sigma-Aldrich, Prague, Czech Republic) as an overlay, supplemented with 20% fetal calf serum (Gibco, Prague, Czech Republic), 1% Basal Medium Eagle vitamins (Sigma-Aldrich, Prague, Czech Republic), 2% sterile urine, and 250 μg/mL amikacin (Amikin, Bristol-Myers Squibb, Prague, Czech Republic). For experimental infections of sand flies, low passage parasites were used, they were washed by centrifugation (2400× *g* for 5 min) and resuspended in sterile saline solution.

Development of different *Leishmania* (*S*.) *tarentolae* strains was studied in three sand fly species occurring in the Mediterranean area. We used laboratory-reared colonies of *Phlebotomus perniciosus* (originating from Spain), *Phlebotomus papatasi*, and *Phlebotomus sergenti* (both originating from Turkey). Sand flies were maintained at 26 °C with a 14 h light/10 h dark photoperiod and fed on 50% sucrose. For a detailed description, see [36].

### 2.2. Sand Fly Infections

Sand fly females (5–9 days old) were infected by feeding through a chick-skin membrane on heat-inactivated sheep blood (LabMediaServis, Jaromer, Czech Republic). Based on preliminary experiments, the infectious dose was set to 5 × 10^6^ promastigotes per 1 mL. Engorged females were separated and maintained under the same conditions as the colonies. Dissections were performed at two time-intervals post blood meal (PBM): on day 1 PBM (before defecation, an early stage of infection) to confirm the experimental blood feeding was successful, and on day 7 PBM (several days after defecation, a late stage of infection). The abundance of parasites and their localization in the sand fly gut were examined under the light microscope. The infections were graded as light (<100 parasites per gut), moderate (100–1000 parasites per gut) and heavy (>1000 parasites per gut), as described previously [37]. All experiments were repeated at least twice for each *Sauroleishmania* strain–sand fly combination. Gut smears of infected sand flies were prepared for morphological forms determination. Differences in intensities of infections were tested by Chi-square test using the software SPSS version 23.

### 2.3. Morphometry of Parasites

Smears of sand fly guts infected by different *Leishmania* (*S*.) *tarentolae* strains were prepared on day 7 post blood meal. Morphometry of promastigotes on day 1 PBM was not performed as the numbers of parasites were too low. Gut smears were fixed with methanol, stained with Giemsa, and examined under the light microscope with an oil-immersion objective. Parasites were photographed with an Olympus D70 camera and measured using ImageJ software. Body length, body width, and flagellar length of 150 randomly selected promastigotes from at least three different sand flies were recorded (for each *Sauroleishmania* strain–sand fly combination). Criteria for morphological forms published by previous authors were modified [21,38,39] and following morphological stages were determined: (i) long nectomonads: body length ≥14 μm; (ii) short nectomonads: body length <14 μm and flagellar length <2 times body length; (iii) metacyclic promastigotes: body length <14 μm and flagellar length ≥2 times body length; (iv) rounded paramastigotes; and (v) haptomonads.

## 3. Results

Development of three *L*. (*S*.) *tarentolae* strains was followed from day 1 to 7 post blood meal (PBM) and all strains showed similar results. On the other hand, *Leishmania* development significantly differed between sand fly species on day 7 PBM (for detailed statistics, see Table S1).

### 3.1. Development of L. (S.) tarentolae in P. papatasi

**Figure 1:**
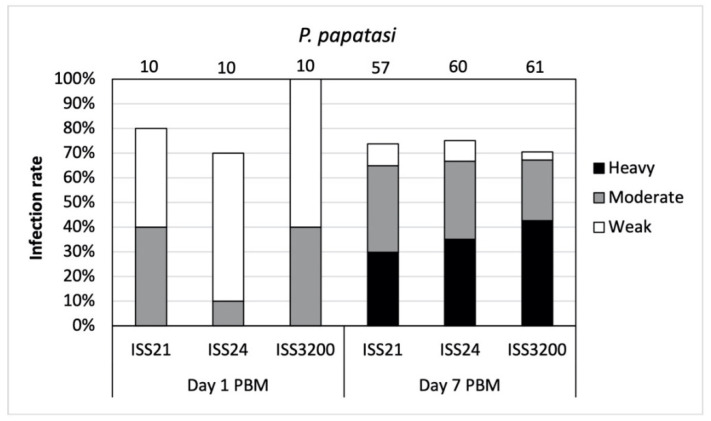
Infection rates and intensities of three *Leishmania* (*S*.) *tarentolae* strains in *Phlebotomus papatasi* on days 1 and 7 post blood meal (PBM). Intensities of infections were classified into three categories: light (<100 parasites/gut), moderate (100–1000 parasites/gut), and heavy (>1000 parasites/gut). Numbers of dissected sand fly females are given above the columns.

In total, 208 *P*. *papatasi* females were dissected. On day 1 PBM, the infection rates ranged from 70–100% for individual *Sauroleishmania* strains. Parasites grew slowly at the beginning and only light or moderate infections were recorded (Figure 1). Promastigotes were present in the endoperitrophic space, inside ingested blood meal surrounded by peritrophic matrix.

On day 7 PBM, fully developed late-stage infections were observed in all three *L*. (*S*.) *tarentolae* strains. Infection rates reached about 70% for each strain and the majority of infections were either heavy or moderate (Figure 1). Statistical differences in the intensities of infections among strains were non-significant (X^2^ = 4.389, df = 6, *p* = 0.624).

**Figure 2:**
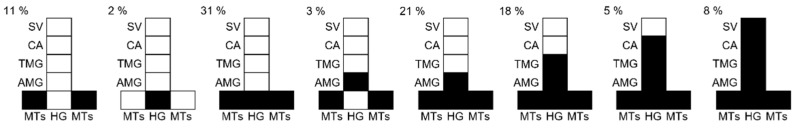
Localization of three *Leishmania* (*S*.) *tarentolae* strains in *Phlebotomus papatasi* on day 7 post blood meal. HG, hindgut; MTs, Malpighian tubules; AMG, abdominal midgut; TMG, thoracic midgut; CA, cardia; SV, stomodeal valve. Percent distribution of localization patterns among the infected females is shown in the top left of each stylized diagram. Only localizations present in more than 1% of females are depicted; for more details about each *Sauroleishmania* strain, see Figure S1.

In the majority (55%) of infected *Phlebotomus papatasi* females, *Sauroleishmania* underwent peripylarian development; promastigotes colonized the hindgut and spread anteriorly to the midgut (Figure 2). Tendency towards an anterior position in the midgut was quite frequent and the stomodeal valve was successfully colonized in 8% of infected females. In the hindgut, both attached (haptomonads) and free-swimming forms (nectomonads) were observed along the entire length of the hindgut. Promastigotes were frequently found also in Malpighian tubules (98% of infected females, see Figure 2) and often were present there in high numbers.

### 3.2. Development of L. (S.) tarentolae in P. sergenti

**Figure 3:**
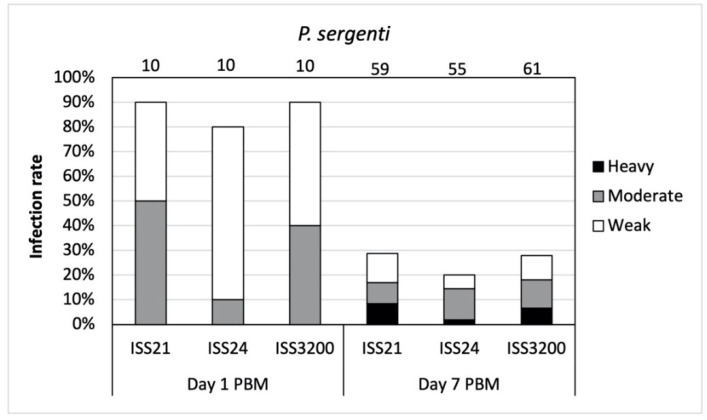
Infection rates and intensities of three *Leishmania* (*S*.) *tarentolae* strains in *Phlebotomus sergenti* on day 1 and day 7 post blood meal (PBM). Intensities of infections were classified into three categories: light (<100 parasites/gut), moderate (100–1000 parasites/gut), and heavy (>1000 parasites/gut). Numbers of dissected sand fly females are given above the columns.

In total, 205 *P*. *sergenti* females were examined for *Sauroleishmania* infections. On day 1 PBM, high infection rates (80–90%) were observed in all three *Sauroleishmania* strains (Figure 3). Nevertheless, the majority of infections were low or moderate, similarly to *Phlebotomus papatasi*. All parasites were localized within the endoperitrophic space.

The development of *L*. (*S*.) *tarentolae* in *P*. *sergenti* was less successful after defecation as infection rates were less than 30% on day 7 PBM. The intensities of infections were mostly light or moderate (Figure 3) and differences among the *Sauroleishmania* strains were not significant (X^2^ = 5.822, df = 6, *p* = 0.443).

**Figure 4:**
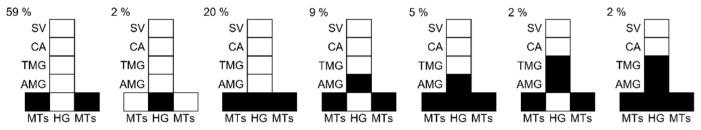
Summarized localization of three *Leishmania* (*S*.) *tarentolae* strains in *Phlebotomus sergenti* on day 7 post blood meal. HG, hindgut; MTs, Malpighian tubules; AMG, abdominal midgut; TMG, thoracic midgut; CA, cardia; SV, stomodeal valve. Percent distribution of localization patterns among the infected females is shown in the top left of each stylized diagram. Only localizations present in more than 1% of females are depicted; for more details about each *Sauroleishmania* strain, see Figure S2.

In *P*. *sergenti* females, most parasites were localized in the Malpighian tubules and the hindgut: 81% of infected females had parasites limited to these two tissues (hypopylarian type of development). Both attached and free forms occurred along the hindgut. In contrast to *P*. *papatasi*, tendency to anterior migration was lower and the peripylarian type of development was recorded only in 18% of infected females: flagellates were observed in abdominal and thoracic parts of the midgut, but never colonized the cardia or the stomodeal valve (Figure 4).

### 3.3. Development of L. (S.) tarentolae in P. perniciosus

**Figure 5:**
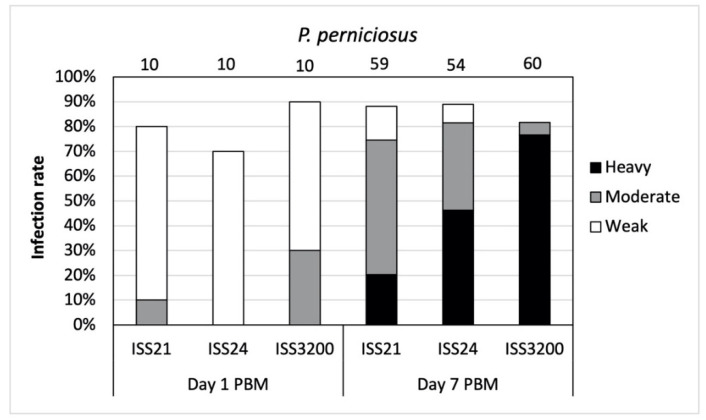
Infection rates and intensities of three *Leishmania* (*S*.) *tarentolae* strains in *Phlebotomus perniciosus* on day 1 and day 7 post blood meal (PBM). Intensities of infections were classified into three categories: light (<100 parasites/gut), moderate (100–1000 parasites/gut), and heavy (>1000 parasites/gut). Number of dissected sand fly females are given above the columns.

In total, 203 *P*. *perniciosus* females were dissected and examined for *Sauroleishmania* infection. On day 1 PBM, the percentage of infected sand flies was relatively high (70–90%) in all *Sauroleishmania* strains. Majority of the infections were light (Figure 5) with all parasites presented in bloodmeal enclosed by the peritrophic matrix.

On day 7 PBM, late-stage infections developed in 80–90% of *P*. *perniciosus*, most of them were moderate or heavy (in all three *Sauroleishmania* strains). Differences in intensities of infections among strains were significant (X^2^ = 52.459, df = 6, *p* = 0.000). In *L*. (*S*.) *tarentolae* strain ISS3200, almost 80% of heavy infections was recorded (Figure 5).

**Figure 6:**
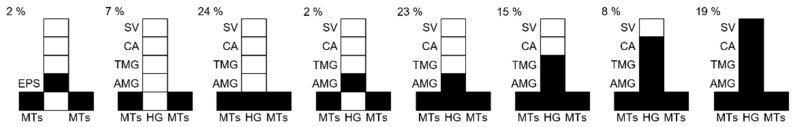
Summarized localization of three *Leishmania* (*S*.) *tarentolae* strains in *Phlebotomus perniciosus* on day 7 post blood meal. EPS, endoperitrophic space; HG, hindgut; MTs, Malpighian tubules; AMG, abdominal midgut; TMG, thoracic midgut; CA, cardia; SV, stomodeal valve. Percent distribution of localization patterns among the infected females is shown in the top left of each stylized diagram. Only localizations present in more than 1% of females are depicted; for more details about each *Sauroleishmania* strain, see Figure S3.

**Figure 7:**
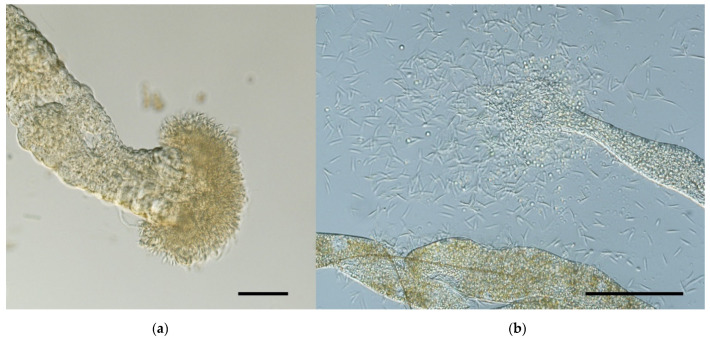
*Phlebotomus perniciosus* infected with *Leishmania* (*S.*) *tarentolae* strain ISS3200 on day 7 post blood meal: (**a**) colonization of stomodeal valve (Nomarski interference contrast, 100× magnification); (**b**) presence of flagellates in Malpighian tubules (Nomarski interference contrast, 200× magnification). Scale bar = 100 μm.

In *P*. *perniciosus*, promastigotes often occupied hindgut, Malpighian tubules, and midgut; their anterior migration was more frequent than in the other two sand fly species tested. Peripylarian type of development clearly prevailed in *P*. *perniciosus*: in two thirds of infected females, parasites were localized in the midgut, and colonization of the stomodeal valve was observed in 19% of females (Figure 6 and Figure 7a). Malpighian tubules were colonized in all infected sand flies (Figure 6) and mostly harbored heavy parasite loads (Figure 7b). In the hindgut, we distinguished both attached and free forms (similarly to *P*. *papatasi* and *P*. *sergenti*).

### 3.4. Morphometry of Promastigotes from Gut Smears

**Figure 8:**
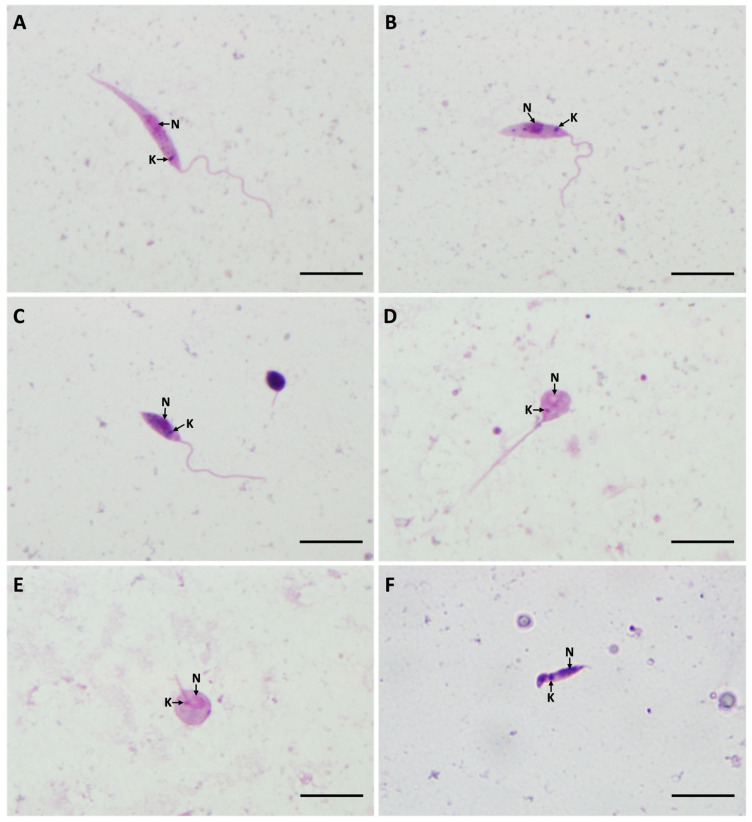
*Leishmania* (*S*.) *tarentolae* morphological forms in *Phlebotomus* sand flies: (**A**) elongated nectomonad; (**B**) short nectomonad; (**C**) metacyclic promastigote; (**D**) rounded metacyclic promastigote; (**E**) rounded paramastigote; (**F**) haptomonad. N, nucleus; K, kinetoplast (stained by Giemsa, 1000× magnification, scale bar = 10 μm).

*Sauroleishmania* morphological forms were studied on day 7 post blood meal in all three sand fly species and compared with those from the culture. In total, 1350 promastigotes from sand fly guts and 450 promastigotes from cultures were photographed, measured, and five morphological forms were distinguished: elongated nectomonads, short nectomonads, metacyclic promastigotes, rounded paramastigotes, and haptomonads. All these forms were seen moving in native preparations from sand fly guts or the cultures and therefore we considered them as typical *Sauroleishmania* developmental stages. For detailed morphometry of individual forms, see the Supplementary Materials (Table S2). The spectrum of morphological forms produced by *L*. (*S*.) *tarentolae* in *Phlebotomus* sand flies is shown in Figure 8.

In the cultures (seven days old, stationary phase of growth), only three types of flagellates were present: elongated nectomonads (16%), short nectomonads, which represented the prevailing forms (64%), and metacyclic promastigotes (20%).

In the sand fly gut, all five types of promastigotes were found. Most prevailing forms were short nectomonads (59%) and elongated nectomonads (26%). Both of these forms were also observed in a variation with significantly shortened flagella, whose average length was around 4.6 μm. The body length of elongated nectomonads was variable and in some parasite cells reached up to 29 μm. The metacyclic promastigotes (14%) were observed in all *Sauroleishmania* strain–sand fly combinations (Table 1) and they appeared in two types, with the short thin body or as rounded metacyclics. Paramastigotes were another morphological form present in the sand fly gut. These forms have small rounded body (~5 μm by 4.5 μm) and very short flagella (~1.2 μm) with the kinetoplast beside the nucleus. Paramastigotes were found in all three sand fly species, but in very low numbers (less than 2% for each). Haptomonads were the least abundant forms found (less than 1%) as they are strongly attached to the cuticular lining of the gut and thus it is hard to detect them on gut smears.

## 4. Discussion

Over the last decades, *Leishmania* (*Sauroleishmania*) *tarentolae* has been commonly used as a model organism due to its biosafety level and easy cultivation of laboratory-adopted strains. This parasite species has made a significant contribution to the study of kinetoplast DNA (kDNA) and RNA editing [40,41,42], it has been used to express human recombinant proteins, and it is also considered for application in the immunotherapy of mammalian leishmaniases [7,11,12,43,44]. In contrast, basic aspects of its development in geckos and sand flies still remain unclear. In this study, we compared the development of various *L*. (*S*.) *tarentolae* isolates in three sand fly species of the genus *Phlebotomus* and demonstrated significant differences in susceptibility of these sand fly species. We are aware that for a better understanding of the *Sauroleishmania* life cycle, it would be important to study the development of *L*. (*S*.) *tarentolae* in its natural vector, *Sergentomyia minuta* [18,33]. A colony of *Se*. *minuta* has recently been established in our laboratory in Prague. Unfortunately, this species is not willing to feed experimentally through any type of membrane (both chicken and gecko skins were tested) and we assume that feeding on infected geckos might be the only possibility how to infect these sand flies.

*Phlebotomus* (*Phlebotomus*) *papatasi* is a common species occurring in the Mediterranean area. It is a specific vector of *Leishmania major* and *Leishmania turanica* [45,46], but is also considered to be potential vector of some *Sauroleishmania* species [17]. Our experiments revealed that all three *L*. (*S*.) *tarentolae* strains were able to develop late-stage infections in this sand fly species. The infection rates were relatively high (about 70% for each strain) with the majority of heavy or moderate parasite loads. *Sauroleishmania* had peripylarian development in the majority of infected females and the stomodeal valve was colonized in 8% of females. These findings are in the agreement with the old study comparing the development of two *L*. (*S*.) *tarentolae* strains in *P*. *papatasi*: in both strains tested, parasites were seen in anterior midgut on days 3 to 5 post infection [21]. Although *P*. *papatasi* feeds primarily on mammals, several studies on host preferences have reported this species being an opportunistic feeder [47,48]. In addition, the ability of *P*. *papatasi* to feed on cold-blooded vertebrates has been repeatedly demonstrated [8,21,49]. Thus, we confirm that these sand flies may play a role in *Sauroleishmania* transmission.

*Phlebotomus* (*Paraphlebotomus*) *sergenti* is a specific vector of *Leishmania tropica* [50] in the Middle East and Maghreb area. According to our study, the development of *L*. (*S*.) *tarentolae* was less successful in this sand fly species, as most of the infections were lost after defecation. In all *Sauroleishmania* strains, less than 30% of dissected females were positive on day 7 post blood meal (PBM) with the majority of weak and moderate infections. In some *P*. *sergenti* females, *Sauroleishmania* underwent a peripylarian type of development, but in majority they were limited to Malpighian tubules and hindgut. The low tendency of anterior migration in *P*. *sergenti* might be due to the inability of parasites to attach to *P*. *sergenti* midgut epithelium as this species is known to be a specific vector of *L*. *tropica* [50]. These findings suggest that *P*. *sergenti* is not a very suitable host for our *L*. (*S*.) *tarentolae* strains. Although *P*. *sergenti* is considered to be an opportunistic species [47,51] and its ability to feed on geckos has been also reported [21], our results suggest that this sand fly species is unlikely to serve as a *L*. (*S*.) *tarentolae* vector.

*Phlebotomus* (*Larroussius*) *perniciosus* is an abundant sand fly species in the western part of the Mediterranean area, a major vector of *Leishmania infantum* [52], and under laboratory conditions it is permissive for several *Leishmania* species [53]. In *P*. *perniciosus*, high infection rates (80–90%) were observed on day 7 PBM in all three *L*. (*S*.) *tarentolae* strains. Most infections were of moderate or heavy intensity, parasites colonized the midgut in two thirds of infected females and 19% of them colonized the stomodeal valve. Our results showed that *P*. *perniciosus* was highly susceptible for all three *L*. (*S*.) *tarentolae* strains studied. Although several studies have reported the broad host range of *P*. *perniciosus* [54,55,56], its willingness to feed on geckos has not been recorded yet. *Leishmania* promastigotes isolated from *P. perniciosus*, even in females collected in Mediterranean sites where *Se. minuta* and *L.* (*S*.) *tarentolae* are sympatric, were repeatedly typed as *L. infantum*, but never as *L.* (*S.*) *tarentolae* [18,52,55,57,58]. However, DNA of *L*. (*S*.) *tarentolae* has recently been detected in *P*. *perniciosus* [15,59] and in another member of subgenus *Larroussius*, *Phlebotomus perfiliewi* [16]. This suggests that more attention should be given to *Larroussius* species as potential secondary vectors of *L*. (*S*.) *tarentolae*.

Members of the subgenus *Sauroleishmania* are traditionally classified as Hypopylaria and their development is localized in the hindgut [22]. Our results showed that in all three *Phlebotomus* species studied, *L*. (*S*.) *tarentolae* occupied both posterior and anterior parts of the midgut and thus had a peripylarian type of development. Peripylarian development prevailed in *P. papatasi* and *P. perniciosus* while it was less frequent in *P. sergenti*. The anterior migration of this *Sauroleishmania* species was also recorded in females of *Sergentomyia minuta* experimentally infected by feeding on gecko *Tarentola mauritanica*, which was positive for mixed infections of *L*. (*S*.) *tarentolae* and *Trypanosoma platydactyli* [17,23].

The occurrence of *L*. (*S*.) *tarentolae* in Malpighian tubules was surprising, as this behavior is more typical for *Endotrypanum* and monoxenous trypanosomatids [60]. In our study, Malpighian tubules (MTs) were colonized in all parasite-vector combinations and often by heavy parasite loads: promastigotes were densely packed in the lumen of MTs. Simultaneous presence of flagellates in blood meal and MTs in few partially defecated females on day 7 PBM suggests that *L*. (*S*.) *tarentolae* enters MTs immediately after the peritrophic matrix is broken. Thus, we suppose that MTs represent the main location for these parasites. Previously, the infection of promastigotes in MTs was recorded only in five specimens of *Se*. *minuta* in the south of France [25]. It was assumed that these sand flies were infected by *L*. (*S*.) *tarentolae*, as this parasite was isolated from geckos *T*. *mauritanica* in the same area [30] and infected *Se*. *minuta* females were collected nearby drainage holes in stone walls where the geckos lived [25]. Our results suggest that the colonization of MTs is a regular part of the life cycle for at least some *Sauroleishmania* species.

There are only few records of *Sauroleishmania* morphological forms in the available literature [21,24,49]. Within this study, we distinguished five morphological forms. On day 7 PBM, the most prevailing forms were elongated nectomonads and short nectomonads. Furthermore, we also observed stages that were morphologically determined as metacyclic promastigotes. In mammalian species, these stages are highly infective for the vertebrate hosts, and they could be distinguished both morphologically and biochemically, as the surface lipophosphoglycan (LPG) is highly modified during the process of metacyclogenesis [61]. However, it should be noted that metacyclogenesis has never been described in *Sauroleishmania* and it is not clear if these forms are infectious for the reptiles. Other less abundant forms we observed were haptomonads and small rounded cells with very short flagella classified as paramastigotes. These rounded forms were previously seen in *P*. *papatasi* experimentally infected with *L*. (*S*.) *ceramodactyli* [21], and therefore we considered them as typical stages of *Sauroleishmania* life cycle in their vectors. As *Sauroleishmania* developmental stages have not been described previously, there is no evidence of the individual forms’ infectivity and further research in this area is needed [26].

Some authors have also described another morphological form typical for *Sauroleishmania*, so-called “fisherman’s floats” [24,49]. These stages have an expanded body in the nucleus area and noticeably attenuated posterior ends. However, “fisherman’s floats” have not been defined in more detail and judging by the authors’ drawings, they are very variable. In our morphological analysis, we sometimes noticed the forms that were quite similar to the “fisherman’s floats”. Nevertheless, we decided not to record them as a specific category as these cells were quite variable and often damaged. Moreover, we did not observe any “fisherman’s floats” in the native preparations and thus we cannot exclude the possibility that forms described on smears by previous authors [24,49] were artifacts resulting from gut smear preparations.

## 5. Conclusions

- We demonstrated the ability of *L*. (*S*.) *tarentolae* to develop in the midgut of three *Phlebotomus* sand flies. The most permissive to parasite development was *P. perniciosus* and we suggest that this species, together with *P. papatasi,* could be involved in *L*. (*S*.) *tarentolae* circulation and should be considered as potential secondary vectors of this parasite.
- *L*. (*S*.) *tarentolae* was frequently found in anterior midgut and stomodeal valve of infected sand fly females, which challenges previous definition of its hypopylarian development (limited to hindgut only). Interestingly, heavy parasite loads were frequently found in Malpighian tubules, which suggests that this localization, found previously in monoxenous *Crithidia* parasites, is unique among *Leishmania* but typical for *L*. (*S*.) *tarentolae* development in *Phlebotomus* sand flies.
- For better understanding of *Sauroleishmania* life cycle, its morphological forms, and localization in the sand fly gut, it would be important to study the development of *L*. (*S*.) *tarentolae* in its proven natural vector *Sergentomyia minuta*.

## Figures and Tables

**Table 1:** Proportion of metacyclic forms developing in culture and in sand flies.

*Sauroleishmania* Strain	Culture	*P. papatasi*	*P. sergenti*	*P. perniciosus*
ISS21	18%	15%	15%	16%
ISS24	21%	12%	12%	12%
ISS3200	22%	11%	15%	16%

## Supplementary Materials

The following are available online at https://www.mdpi.com/article/10.3390/microorganisms9112256/s1, Table S1: Comparison of intensities of late-stage infections between three sand fly species. Figure S1: Detailed localization of three *Leishmania* (*S*.) *tarentolae* strains (ISS21, ISS24, and ISS3200) in *Phlebotomus papatasi* on day 7 post blood meal. Figure S2: Detailed localization of three *Leishmania* (*S*.) *tarentolae* strains (ISS21, ISS24, and ISS3200) in *Phlebotomus sergenti* on day 7 post blood meal. Figure S3: Detailed localization of three *Leishmania* (*S.*) *tarentolae* strains (ISS21, ISS24, and ISS3200) in *Phlebotomus perniciosus* on day 7 post blood meal. Table S2: Detailed morphometry in microns of individual forms of *Leishmania* (*S*.) *tarentolae*.
