# Supplementary material for: Development of Various Leishmania (Sauroleishmania) tarentolae Strains in Three Phlebotomus Species
Source: Microorganisms. 2021 Oct 29;9(11):2256. doi: 10.3390/microorganisms9112256 (PMC8622532; doi:10.3390/microorganisms9112256)
Supplement: Supplementary file 1 [file microorganisms-09-02256-s001.zip › Figure S1.pdf]

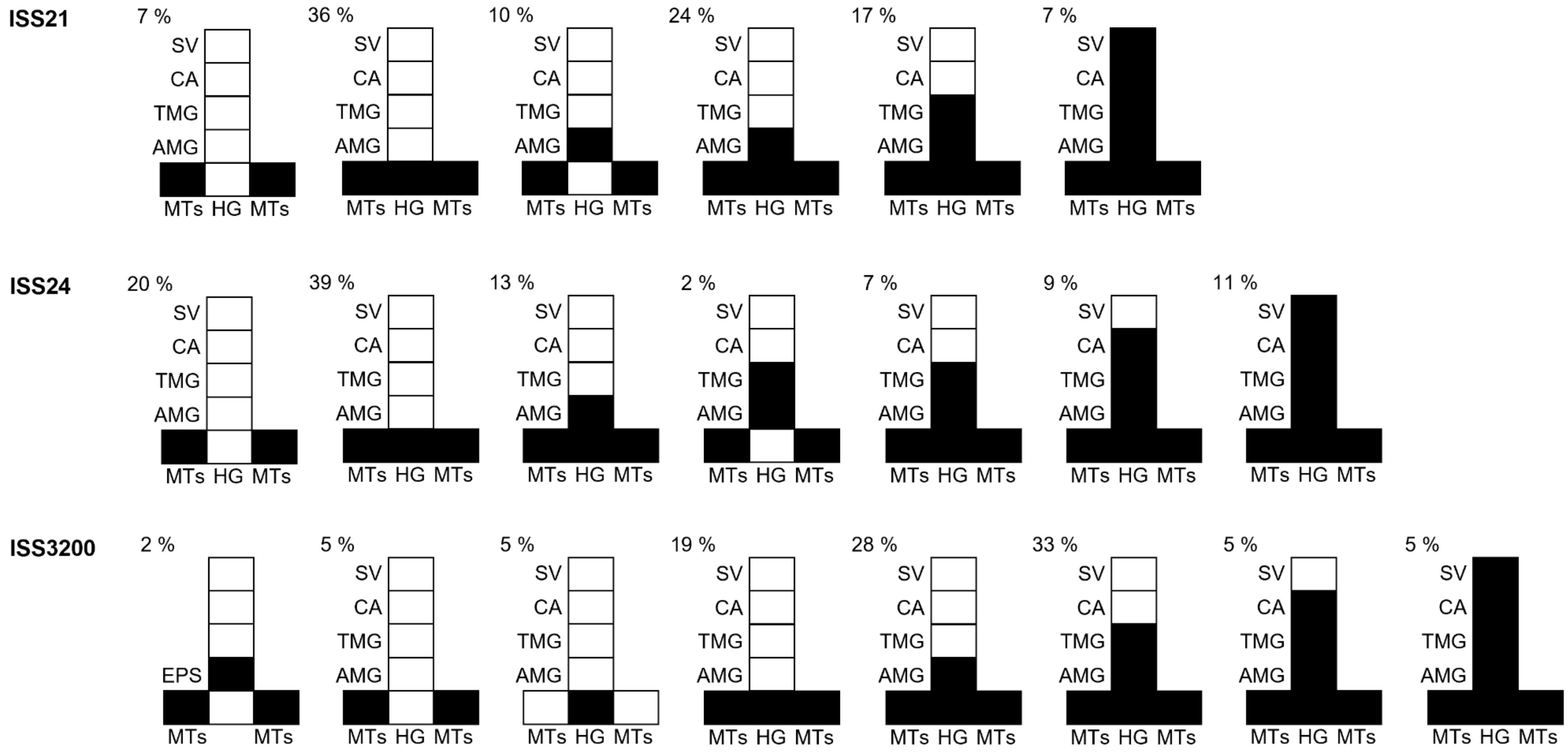

Figure S1: Detailed localization of three *Leishmania* (*S.*) *tarentolae* strains (ISS21, ISS24, and ISS3200) in *Phlebotomus papatasi* on day 7 post blood meal. EPS, endoperitrophic space; HG, hindgut; MTs, Malpighian tubules; AMG, abdominal midgut; TMG, thoracic midgut; CA, cardia; SV, stomodeal valve. Percent distribution of localization patterns among the infected females is shown in the top left of each stylized diagram.
