# Supplementary material for: Development of Various Leishmania (Sauroleishmania) tarentolae Strains in Three Phlebotomus Species
Source: Microorganisms. 2021 Oct 29;9(11):2256. doi: 10.3390/microorganisms9112256 (PMC8622532; doi:10.3390/microorganisms9112256)
Supplement: Supplementary file 1 [file microorganisms-09-02256-s001.zip › Table S1.pdf]

Table S1. Comparison of intensities of late-stage infections between three sand fly species.

Sauroleishmania strain	Sand fly species	N	Statistics
ISS21	*P. papatasi*	57	X^2 = 57.385, *df* = 6, *P* = 0.000
	P. sergenti	59	
	P. perniciosus	59	
ISS24	*P. papatasi*	60	X^2 = 67.627, *df* = 6, *P* = 0.000
	P. sergenti	55	
	P. perniciosus	54	
ISS3200	*P. papatasi*	61	X^2 = 75.728, *df* = 6, *P* = 0.000
	P. sergenti	61	
	P. perniciosus	60	
