# Supplementary material for: Development of Various Leishmania (Sauroleishmania) tarentolae Strains in Three Phlebotomus Species
Source: Microorganisms. 2021 Oct 29;9(11):2256. doi: 10.3390/microorganisms9112256 (PMC8622532; doi:10.3390/microorganisms9112256)
Supplement: Supplementary file 1 [file microorganisms-09-02256-s001.zip › Table S2.pdf]

Table S2. Detailed morphometry in microns of individual forms of *Leishmania* (*S.*) *tarentolae*.

Morphological forms		Body length	Body width	Flagellar length
Elongated nectomonads	N	420	420	420
	Mean	16,277	2,466	19,635
	Median	15,800	2,400	19,700
	Minimum	14,0	1,2	3,0
	Maximum	29,2	5,0	32,9
	Std. Deviation	2,0216	0,4724	3,9314
Metacyclic promastigotes	N	192	192	192
	Mean	8,172	2,553	18,904
	Median	7,900	2,500	18,800
	Minimum	4,9	1,1	10,1
	Maximum	13,1	5,2	30,6
	Std. Deviation	1,6401	0,7147	3,7813
Short nectomonads	N	1081	1081	1081
	Mean	10,506	2,396	14,770
	Median	10,700	2,300	14,900
	Minimum	4,0	1,0	1,9
	Maximum	13,9	6,5	26,0
	Std. Deviation	2,1007	0,5956	3,5724
Rounded metacyclic promastigotes	N	85	85	85
	Mean	5,494	4,299	16,715
	Median	5,500	4,300	16,300
	Minimum	3,2	2,8	8,9
	Maximum	8,1	7,5	26,6
	Std. Deviation	1,0607	0,9348	3,9173
Paramastigotes	N	19	19	19
	Mean	5,232	4,589	1,232
	Median	5,300	4,900	1,300
	Minimum	3,1	2,5	0,4
	Maximum	7,0	5,8	2,1
	Std. Deviation	1,0425	0,9146	0,4933
Haptomonads	N	3	3	3
	Mean	8,767	2,400	2,167
	Median	6,800	2,400	2,400
	Minimum	6,1	2,2	1,5
	Maximum	13,4	2,6	2,6
	Std. Deviation	4,0278	0,2000	0,5859
Total	N	1800	1800	1800
	Mean	11,309	2,542	16,274
	Median	11,000	2,400	16,300
	Minimum	3,1	1,0	0,4
	Maximum	29,2	7,5	32,9
	Std. Deviation	3,6285	0,7567	4,5787
